# Oxidative Fermentation of Acetic Acid Bacteria and Its Products

**DOI:** 10.3389/fmicb.2022.879246

**Published:** 2022-05-24

**Authors:** Yating He, Zhenzhen Xie, Huan Zhang, Wolfgang Liebl, Hirohide Toyama, Fusheng Chen

**Affiliations:** ^1^Hubei International Scientific and Technological Cooperation Base of Traditional Fermented Foods, Huazhong Agricultural University, Wuhan, China; ^2^College of Food Science and Technology, Huazhong Agricultural University, Wuhan, China; ^3^Department of Microbiology, Technical University of Munich, Freising, Germany; ^4^Department of Bioscience and Biotechnology, Faculty of Agriculture, University of the Ryukyus, Okinawa, Japan

**Keywords:** acetic acid bacteria, classification, membrane-bound dehydrogenase, oxidative fermentation, molecular biology

## Abstract

Acetic acid bacteria (AAB) are a group of Gram-negative, strictly aerobic bacteria, including 19 reported genera until 2021, which are widely found on the surface of flowers and fruits, or in traditionally fermented products. Many AAB strains have the great abilities to incompletely oxidize a large variety of carbohydrates, alcohols and related compounds to the corresponding products mainly including acetic acid, gluconic acid, gulonic acid, galactonic acid, sorbose, dihydroxyacetone and miglitol *via* the membrane-binding dehydrogenases, which is termed as AAB oxidative fermentation (AOF). Up to now, at least 86 AOF products have been reported in the literatures, but no any monograph or review of them has been published. In this review, at first, we briefly introduce the classification progress of AAB due to the rapid changes of AAB classification in recent years, then systematically describe the enzymes involved in AOF and classify the AOF products. Finally, we summarize the application of molecular biology technologies in AOF researches.

## Introduction

Acetic acid bacteria (AAB) are a group of Gram-negative, strictly aerobic bacteria within the family Acetobacteraceae (Saichana et al., 2015), which habitat in a large variety of different sources, such as flowers or fruits (Trček and Barja, 2015), guts of some insects (Crotti et al., 2016), and various traditionally fermented foods including vinegar, lambic beer, kefir, kombucha and so on (De Roos and De Vuyst, 2018). AAB may be named after their abilities to produce acetic acid *via* ethanol oxidation (Nanda et al., 2001), but actually some AAB strains are unable to produce acetic acid from ethanol, such as some strains within AAB genera of *Asaia* (*As.)* and *Saccharibacter* (*Sa*.; Moore et al., 2002; Jojima et al., 2004). Meanwhile, some AAB strains can fix nitrogen (Fuentes-Ramírez et al., 2001), produce pigment (Malimas et al., 2009), or exopolysaccharide (EPS; Gullo et al., 2017; La China et al., 2018). Very importantly, many AAB strains can incompletely oxidize various carbohydrates, alcohols and related compounds to yield the corresponding industrial products such as acetic acid, gluconic acid (GA), galactonic acid, 2-keto-l-gulonic acid (2-KGA), dihydroxyacetone (DHA), miglitol and so on (Mamlouk and Gullo, 2013), which have been successfully used in foods, cosmetics, medicines and other fields (Gullo et al., 2014; Saichana et al., 2015). These partial oxidation processes of AAB are named as AAB oxidative fermentation (AOF).

**Figure 1:**
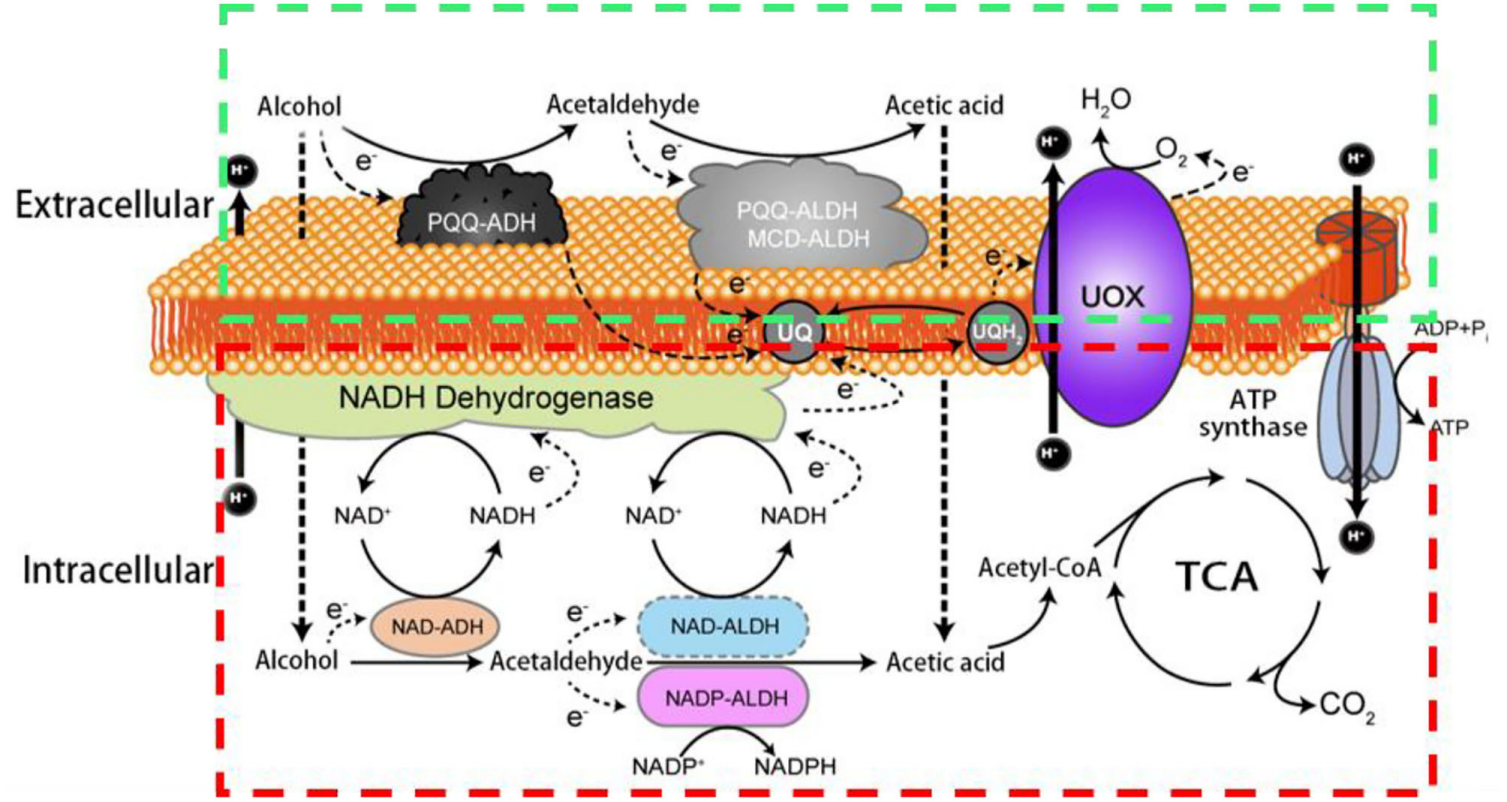
Schematic diagram of two respiratory chains of alcohol in acetic acid bacteria. PQQ-ADH: pyrroloquinoline quinone dependent alcohol dehydrogenase; PQQ-ALDH: pyrroloquinoline quinone dependent acetaldehyde dehydrogenase; MCD-ALDH: molybdenum-molybdopterin cytosine dinucleotide dependent acetaldehyde dehydrogenase; NAD-ADH: nicotinamide adenine dinucleotide dependent alcohol dehydrogenase; NAD- ALDH: nicotinamide adenine dinucleotide dependent acetaldehyde dehydrogenase; NADP-ALDH: nicotinamide adenine dinucleotide phosphate dependent acetaldehyde dehydrogenase; UQ: ubiquinone; UQH2: ubiquinol; UOX: ubiquinol oxidase; ATP: the energy; TCA: tricarboxylic acid cycle; The green box shows the AOF respiratory chain including PQQ-ADH, PQQ- ALDH (MCD-ALDH), UQ, UQH2; UOX and ATP synthase; the red box indicates the common respiratory chain including NAD-ADH, NAD-ALDH (NADP-ALDH), UQ, UQH2; UOX and ATP synthase.

In AOF, the membrane-binding dehydrogenases (mDH) localized on the periplasmic side of the cytoplasmic membrane of AAB can deprive electrons from the substrates, followed by transferring them to ubiquinone (UQ, also called as coenzyme Q), which is then reduced to ubiquinol (UQH_2_), and eventually, the terminal oxidases (TO) transfer the electrons from UQH_2_ to oxygen to produce UQ, H_2_O, and the energy (ATP; Adachi et al., 2003; Matsushita et al., 2016; Zhang and Chen, 2022). Therefore, besides the common electronic respiratory chain which is located in the bacterial cell membrane, another special respiratory chain (hereinafter referred to as AOF respiratory chain) also co-exist in AAB cells, and make them rapidly get the energy released from AOF (Matsushita et al., 1994, 2004; Yakushi and Matsushita, 2010). The AAB strains with oxidative fermentation capacity are called as oxidative bacteria. It is worth mentioning that the oxidative fermentation and its corresponding respiratory chain not only exist in AAB cells, but also in other aerobic bacteria such as *Pseudomonas* spp. and *Enterobacter* spp. (Matsushita et al., 2016). In Figure 1 the two respiratory chains of alcohol (ethanol) in AAB are showed.

Since AOF takes place on the periplasmic side of the cytoplasmic membrane of AAB, its products are directly released extracellular, and avoid the transport limitation from intracellular to extracellular, resulting that AAB cells are considered as very ideal biocatalysts to produce corresponding products (Matsushita et al., 2016; Sengun, 2017). In this review, we have collected 86 AOF products which have been published, and introduced the relative enzymes with AOF. In addition, we have summarized the advance of the AAB classification and molecular biotechnology.

## AAB Classification Advance

The first AAB genus, *Acetobacter* (*A*.) was proposed by Beijerinck in 1898 (Wang and Chen, 2014). It was not until 37 years later (1935) that the second AAB genus, *Gluconobacter* (*G*.) was described by Asai (1968) and Yamada and Yukphan (2008). By the year of 1989, only 3 genera and 10 species of AAB were generally recognized (Wang and Chen, 2014). Since then the discovery and identification of AAB genera and species have been achieving rapid progress thanks to the development of molecular biology techniques (Wang and Chen, 2014). At the end of 2021, 19 genera and 110 species of AAB have been reported (https://lpsn.dsmz.de/; Supplementary Table 1).

The early classification and identification of AAB were mainly based on the phenotypic traits such as colony and microscopic morphologies, catalase test, Gram staining, chemotaxonomical characteristics mainly including UQ types (UQ9 or UQ10) and the profile of fatty methyl esters in AAB cells. For example, in Yamada and Kondo (1984) divided the genus of *Acetobacter* into two subgenera: *Acetobacter* subgenus with UQ9 and *Gluconoacetobacter* (*Ga*.) subgenus with UQ10, and Urakami et al. (1989) combined phenotypic characteristics, UQ types and the profile of fatty methyl esters to establish a new AAB genus—*Acidomonas* (*Ac*.), which was the third AAB genus recognized at that time.

In recent decades, with the development of molecular biology technologies, especially those related to rRNA, the AAB classification has developed very quickly, leading that some former AAB species or genera have excluded of the AAB group, meanwhile some new species and genera of AAB are proposed or independent from the former AAB (sub) species or (sub)genera. For instance, based on the phylogenic tree of 16S rRNA gene sequences, the genus *Gluconacetobacter*, which once belonged to the subgenus of *Acetobacter*, was elevated to the genus level (Yamada et al., 1997). Later, Yamada and his colleagues discovered that the 16S rRNA gene phylogenetic tree indicated two subclusters within the genus *Gluconacetobacter*, resulting that a new AAB genus *Komagataeibacter* (*K*.) was independent from the genus *Gluconacetobacter* (Yamada and Yukphan, 2008; Yamada et al., 2012a,b), and *Ga. kakiaceti, Ga. Medellinesis*, and *Ga. maltaceti* were re-classified into *K. kakiaceti, K. medellinesis*, and *K. maltaceti*, respectively (Yamada, 2014).

In Table 1, we have summarized the classification changes of AAB genera and species in recent decades.

**Table 1:** The changes of classification status of AAB genera and species in recent decades.

**Current species names**	**Once used species names**	**References**
*Acidomonas* (*Ac*.) *methanolica*	*A. methanolicus*	Urakami et al., 1989
*Frateuria aurantia* (not AAB)	*A. aurantius*	Swings et al., 1980
*Ga. diazotrophicus*	*diazotrophicus, G. diazotrophicus*	Gillis et al., 1989; Yamada et al., 1997
*Ga. liquefaciens*	*A. liquefaciens, G.liquefaciens*	Yamada et al., 1997
*G. japonicas*	*G. industrius, G. nephelii*	Malimas et al., 2008
*G. oxydans*	*G. suboxydans, G. uchimurae, G. melanogenus*	Gosselé et al., 1983; Li et al., 2017
*G. sphaericus*	*G. oxydans* subsp. *sphaericus*	Malimas et al., 2008
*G. thailandicus*	*G. suboxydans, G. oxydans*	Tanasupawat et al., 2004
*Ketogulonicigenium vulgare* (not AAB)	*G. oxydans*	Urbance et al., 2001
*K. europaeus*	*A. europaeus, Ga. europaeus*	Yamada et al., 2012a
*K. hansenii*	*A. hansenii, Ga. hansenii*	Yamada et al., 2012a
*K. intermedius*	*A.intermedius, Ga. Intermedius*	Yamada et al., 2012b
*K. kakiaceti*	*Ga. kakiaceti*	Yamada, 2014
*K. kombuchae*	*Ga. kombuchae, Ga. hansenii*	Yamada et al., 2012a
*K. maltaceti*	*Ga. Maltaceti*	Yamada, 2014
*K. medellinensis*	*Ga. xylinus, Ga. medellinensis*	Marič et al., 2020
*K. nataicola*	*Ga. nataicola*	Yamada et al., 2012a
*K. oboediens*	*A. oboediens, Ga. oboediens*	Yamada, 2000; Yamada et al., 2012b
*K. rhaeticus*	*Ga. Rhaeticus*	Yamada et al., 2012b
*K. saccharivorans*	*Ga. saccharivorans*	Yamada et al., 2012b
*K. sucrofermentans*	*A. xylinum* subsp. *sucrofermentans, Ga. sucrofermentans*	Yamada et al., 2012a
*K. swingsii*	*A. xylinum* subsp. *nonacetooxidans, Ga. Swingsii*	Yamada et al., 2012b
*K. xylinus*	*A. xylinus, A. aceti* subsp. *xylinum, Ga. xylinus*	Yamada et al., 1997, 2012a,b

*A., Acetobacter; Ac., Acidomonas; G., Gluconobacter; Ga., Gluconoacetobacter; K., Komagataeibacter*.

However, only adopting the molecular biology methods related to rRNA may bring an error in AAB classification and identification. For example, according to the 16S rRNA phylogenic tree, the AAB genera of *Asaia* (*As*.), *Kozakia* (*Ka*.), *Swaminathania* (*Sa*.), and *Neoasaia* (*N*.) (Supplementary Table 1) could be categorized as one single genus, but their phenotypes warranted their distinction on the different genus level (Kersters et al., 2006). Therefore, internal transcribed spacer (ITS) of 16S-23S rRNA gene, restriction fragment length polymorphisms of genomic DNA and DNA-DNA hybridization (Janda and Abbott, 2007; Trček and Barja, 2015; Yamada, 2016) were also applied in AAB taxonomic studies. Moreover, the sequences of some genes were applied to identify AAB. For example, Trcek et al. (2006) used the nucleotide sequence of gene *adhA* for AAB identification, while genes of *nifD* and *nifH* were applied to identify nitrogen-fixing AAB species (Loganathan and Nair, 2004; Dutta and Gachhui, 2006). And multilocus sequence analysis of the three genes (*dnaK, groEL*, and *rpoB*) was performed to differentiate AAB species (Cleenwerck et al., 2010).

In the future, in order to obtain more objective, precise and reliable AAB classification, there is no doubt that a multidimensional method of the morphological classification combined with multiple molecular biological methods such as different genes or/ and the complete genome comparison, should be utilized for the classification and identification of AAB strains (Wang and Chen, 2014; Yamada, 2016).

## AAB Oxidative Fermentation

Due to their long existence in sugar-rich environments such as fruits and flowers, some AAB strains are adaptively evolved their abilities to rapidly incompletely oxidize sugars, sugar alcohols, or/and alcohols by mDH to produce corresponding products like aldehydes, ketones, acids, and other products, and yield ATP *via* the AOF respiratory chain (Figure 1; Matsushita et al., 1994, 2002; Saichana et al., 2015).

In addition to AOF, AAB can also completely oxidize sugars, sugar alcohols, alcohols, organic acids, and other substances to CO_2_ and H_2_O through the Embden–Meyerhof–Parnas pathway, the tricarboxylic acid cycle, the pentose phosphate pathway, the Entner–Doudoroff pathway, and/or the glyoxylate pathway, and produce intermediates for cell growth and ATP through the common respiratory chain (Figure 1). Although both complete and incomplete oxidation systems simultaneously exist in AAB cells, usually two systems rarely show similar activities in the same growth period of AAB strains. In an environment of a high concentration of sugars, alcohols, and/or acids, AAB cells mainly carry out AOF in the early growth period and complete oxidation in the late growth period. Aldehydes, ketones, or/and acids accumulated *via* AOF can inhibit the growth of other microorganisms, subsequently, when substrates are almost consumed by AOF, AAB can utilize these AOF products to continue to grow through the complete oxidation, resulting in AAB cells possessing the good growth and survival competitiveness. From the view of ecological evolution, AOF should be considered as a unique characteristic for AAB to adapt to the growth and survival environments, so it leading the carbon sources used by AAB is very complex, especially when multiple carbon sources such as sugars and alcohols are simultaneously present in the media (Gupta et al., 2001; Deppenmeier et al., 2002; Adachi et al., 2003; Raspor and Goranovič, 2008; Mamlouk and Gullo, 2013; Nishikura-Imamura et al., 2014; Saichana et al., 2015). The strategies of AAB to cope with changes in their growth and survival environments (culture conditions) by adjusting AOF were summarized in the reference (Qin et al., 2022).

Since the dehydrogenases (DHs) involved in AOF are located in the cell membrane, they are called as mDHs, while DHs in the cytoplasm is called as cytoplasmic DHs (cDHs). When AAB are exposed to a high concentration of substrate such as sugars and/or alcohols, the DH activity in AAB cells is mainly reflected by mDH, whereas cDH is almost inactive, but with the decrease of concentration of substrates, the cDH activity gradually increases, while the mDH activity decreases or hardly functions (Baldrian, 2006; Hölscher and Görisch, 2006). There are great differences in the coenzymes between mDHs and cDHs, mDHs ones are quite diverse, mainly including pyrroloquinoline quinone (PQQ), molybdenum-molybdopterin cytosine dinucleotide (MCD), flavin adenine dinucleotide (FAD), nicotinamide adenine dinucleotide (NAD), or/and nicotinamide adenine dinucleotide phosphate (NADP; Table 2), while cDHs ones mainly include NAD or/and NADP (Figure 1; Matsushita et al., 1994). The electrons and protons (H^+^) from the substrate are deprived by both mDHs and cDHs, and transferred to UQ to produce UQH_2_, which is then oxidized to produce a proton potential between intracellular and extracellular by terminal oxidase (TO), thereby driving ATP synthase to yield ATP (Figure 1).

**Table 2:** The membrane-binding dehydrogenases in acetic acid bacteria based on their coenzyme differences.

**Category**	**Enzyme and code number**	**Substrate**	**Product**	**Subunit^**a**^**	**Prosthetic group1^**b**^**	**Prosthetic group2^**b**^**	**Electron acceptor^**c**^**
Quinoprotein-cytochrome complex	mADH (EC 1.1.1.1)	Ethanol	Acetaldehyde	I-II-III or I-II	PQQ	4 heme *c*	UQ
Molybdoprotein-cytochrome complex	mALDH (EC 1.2.1.10)	Acetaldehyde	Acetic acid	II-III or I-II	MCD or PQQ	[2Fe-2S] and 3 heme *c*; [2Fe- 2S] and heme *b*; or heme *b* and *c*	UQ*
Flavoprotein-cytochrome complex	mGADH (EC 1.1.99.3)	d-Gluconic acid	2-KGA	I-II-III	FAD	3 heme *c*	UQ
	m2-KGDH (EC 1.1.99.4)	2-KGA	2, 5-DKGA	I-II-III	FAD	3 heme *c*	UQ*
	mFDH (EC 1.1.99.11)	d-Fructose	5-KF	I-II-III	FAD	3 heme *c*	UQ*
	mSLDH (EC 1.1.99.21)	d-Sorbitol	l-Sorbose	I-II-III	FAD	3 heme *c*	UQ*
Membrane-binding quinoprotein	mGDH (EC 1.1.99.17)	d-Glucose	GAL	I-II	PQQ	—^d^	UQ
	mGLDH (EC 1.1.1.6)	Polyalcohol	Ketone	I-II	PQQ	—	UQ
	mQDH (EC 1.1.99.25)	Quinic acid	3-DQA	I-II	PQQ	—	UQ
	mIDH (EC 1.1.1.18)	Myo-inositol	2-Keto-myoinositol	I-II	PQQ	—	UQ*
Other	mSDH (EC 1.1.99.12)	l-Sorbose	l-Sorbone	—^**4**^	FAD	—	UQ
	mSNDH (EC 1.1.1.-)	l-Sorbosone	2-KGLA	—	NAD or NADP or PQQ	—	UQ*

a *I-II-III: three subunits (large, medium-sized and small) complex; I-II: two subunits (large and medium-sized) complex.*

b *Prosthetic groups 1 and 2 are involved in substrate oxidation and electron transfer.*

c UQ is experimentally verified by the experiments, and UQ^*^ means that it has not been experimentally verified.

d *“—”: indicates that no such prosthetic group or subunit complex exists.*

*mADH, membrane-binding alcohol dehydrogenase; mALDH, membrane-binding acetaldehyde dehydrogenase; mGADH, membrane-binding gluconate dehydrogenase; m2-KGDH, membrane-binding 2-keto-d-gluconate dehydrogenase; mFDH, membrane-binding d-fructose dehydrogenase; mSLDH, membrane-bindingd-sorbitol dehydrogenase; mGDH, membrane-binding glucose dehydrogenase; mGLDH, membrane-binding glycerol dehydrogenase; mQDH, membrane-binding quinic acid dehydrogenase; mIDH, membrane-binding inositol dehydrogenase; [2Fe-2S], 2 iron-sulfur clusters; mSDH, membrane-binding sorbose dehydrogenase; mSNDH, membrane-binding sorbone dehydrogenase; 2-KGA, 2-keto-d-gluconic acid; 2, 5-DKGA, 2, 5-diketo-d-gluconic acid; 5-KF, 5-keto-d-fructose; GAL, gluconic acid-δ-lactone; 3-QDA, 3-dehydroquinic acid; 2-KGLA, 2-keto-l-gulonic acid; PQQ, pyrroloquinoline quinone; MCD, molybdenum-molybdopterin cytosine dinucleotide; FAD, flavin adenine dinucleotide; NAD, nicotinamide adenine dinucleotide; NADP, nicotinamide adenine dinucleotide phosphate; UQ, ubiquinone*.

Based on the coenzyme difference, the AAB mDH can be divided into five categories: quinoprotein-cytochrome complex, molybdoprotein-cytochrome complex, flavoprotein– cytochrome complex, quinoprotein, and others (Table 2). The mDH's types and characteristics vary in different genera, species, or strains of AAB. For example, membrane-binding alcohol dehydrogenase (mADH) from the genus *Gluconobacter* can oxidize ethanol into acetic acid, and can also oxidize d-glucose, GA, d-sorbitol, and glycerol into the corresponding products. In contrast, mADH from the genus *Acetobacter* or *Komagataeibacter* can only oxidize ethanol, almost impossible to oxidize other substrates (Matsushita et al., 2016).

TO is another key enzyme in the AOF respiratory chain, which can transfer electrons and protons from UQH_2_ to O_2_ to generate H_2_O_2_ or H_2_O. The TOs from AAB and other aerobic bacteria can be divided into heme-copper oxidase (HCO) and heme *bd* type oxidase (HBD-O). Among them, HCO includes cytochrome *c* oxidase (COX) which can accept electrons from cytochrome *c*, and ubiquinol oxidase (UOX) which can accept electrons from UQH_2_, and have binuclear O_2_-reducing sites consisting of heme *a, o* or/and *b* and one copper atom (Matsutani et al., 2014). Bacteria with COX are called “oxidase-positive” bacteria, such as strains from the genera of *Paracoccus* and *Pseudomonas*, while bacteria with UOX are called “oxidase-negative” bacteria, such as *Escherichia coli* and AAB strains. In addition, HBD-O of AAB is also one kind of UOX that can accept electrons from UQH_2_, but its oxygen reduction site contains heme *b* and *d*, which has a strong affinity for oxygen and can perform aerobic respiration under low oxygen conditions. Moreover, HBD-O has no proton pump function but has a certain proton release capacity that can produce part of the proton potential. In a word, the TOs of AAB mainly include UOX and HBD-O (Qin et al., 2022).

### Membrane-Binding Dehydrogenase (mDH) in AOF

**Figure 2:**
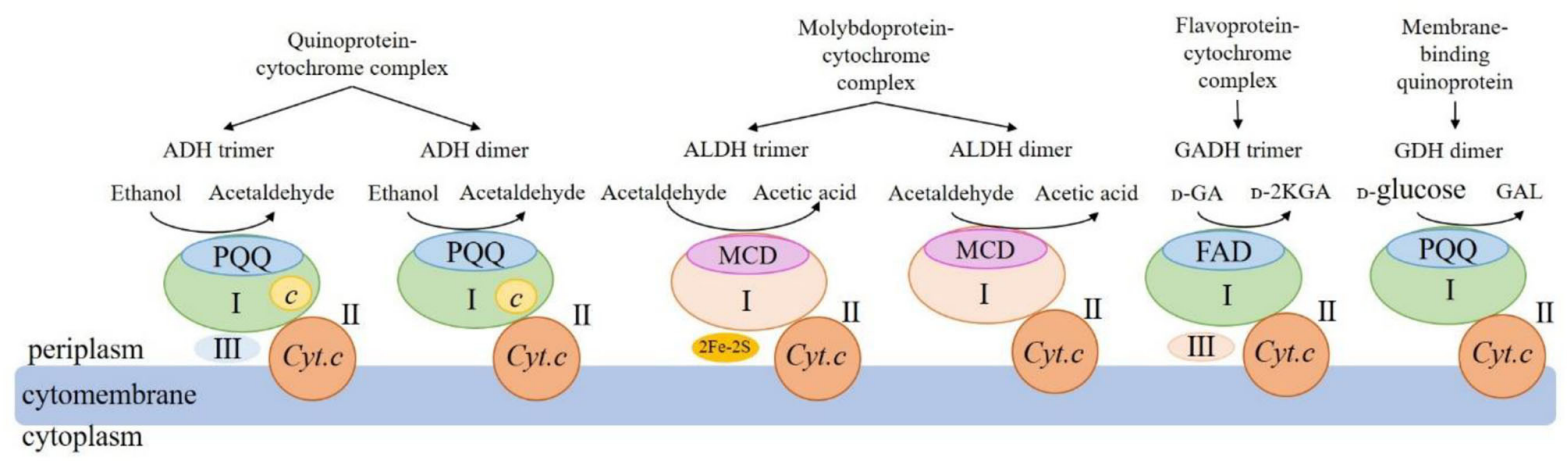
Schematic of structures and functions of the four main membrane-binding dehydrogenases from acetic acid bacteria. ADH: membrane-binding alcohol dehydrogenase; ALDH: membrane-binding acetaldehyde dehydrogenase; GADH: membrane-binding gluconate dehydrogenase; GDH: membrane-binding glucose dehydrogenase; PQQ: pyrroloquinoline quinone; MCD: molybdenum-molybdopterin cytosine dinucleotide; FAD: flavin adenine dinucleotide; c: heme c; Cyt.c: cytochrome c; UQ: ubiquinone; UQH2: reduced ubiquinone; 2Fe-2S: 2 iron-sulfur clusters; I: large subunits; II: medium-size subunits; III: small subunits; GA: gluconic acid; 2-KGA: 2-keto-D-gluconic acid; GAL: gluconic acid-ä-lactone.

mDHs involved in the AOF drive functions are usually present in the form of heterotrimer or heterodimer. The structures and functions for some mDHs from AAB cells that have been clearly studied at present are shown in Figure 2.

#### Quinoprotein–Cytochrome Complex

The mDH of quinone protein-cytochrome complex clearly studied at present is mADH [EC.1.1.1.1], which is a constitutive ethanol ubiquinone oxidoreductase, and can catalyze the oxidation of ethanol to aldehyde and the reduction of UQ to UQH_2_ (Table 2). The mADH isolated from the cell membrane of *Gluconobacter* spp. consists of three subunits: the large subunit (I) containing one PQQ and a heme *c* binding-sites, respectively, can oxidize ethanol to acetaldehyde; the cytochrome *c* subunit (II) including three heme *c* binding sites, can reduce UQ to UQH_2_; and the small subunit (III) without any coenzyme binding site may be involved in cell membrane binding (Adachi et al., 2007; Masud et al., 2010). However, mADHs from *K. europaeus* and *A. peroxydans* (currently *A. pasteurianus*) contain only subunits I and II, no subunit III (Tayama et al., 1989; Trcek et al., 2006). When the dissociation of subunits I and II, the mADH enzyme activity of subunit I decrease significantly, but its enzyme activity is recovered after it re-combinates with subunit II, indicating that the complex of subunits I and II is necessary to maintain the mADH activity. The mADH contains a high-affinity UQ binding site and a catalytic site for the UQ oxidation-reduction enzyme activity, and UQ is involved in electron transfer among heme *c*, UQ, and UQH_2_. In summary, based on the current research results, the AAB mADH is a heterotrimer (I-II-III) or a dimer (I-II) membrane-binding quinoprotein-cytochrome complex and includes prosthetic groups: PQQ and heme *c* (Table 2 and Figure 2).

The substrate specificity of AAB mADH usually is very poor. Except for methanol, short-chain alcohols such as ethanol, 1-propanol, 1-butanol, 1-pentanol, and 1-hexanol can be utilized as its substrates (Shinagawa et al., 2006). In addition, mADH can also produce glyceraldehyde from glycerol, although it only has a low affinity for glycerol. Especially when the glycerol concentration is greater than 10% (W/V), its ability to oxidize glycerol is significantly improved (Habe et al., 2009). Moreover, aldehydes can be applied as substrates of mADH, too, and their oxidation rates by mADHs are almost same as those of the corresponding alcohols (Gómez-Manzo et al., 2008). Therefore, mADH alone can carry out the entire oxidation process from ethanol to acetaldehyde to acetic acid without the involvement of membrane-binding acetaldehyde dehydrogenase (mALDH) [EC 1.2.1.10] (Gómez-Manzo et al., 2015). In addition, although mADH cannot oxidize methanol, it can oxidize formaldehyde so that mADH can be developed as a formaldehyde scavenger (Shinagawa et al., 2006).

#### Molybdoprotein–Cytochrome Complex

The well-studied molybdoprotein-cytochrome complex mDH at present is mALDH, which is an acetaldehyde ubiquinone oxidoreductase. It is generally considered to be a heterotrimer (I-II-III) consisting of a large subunit (I) with MCD as one coenzyme, a medium-size subunit (II) with three heme *c* as prosthetic groups, and a small subunit (III) with two iron-sulfur clusters [2Fe-2S] as prosthetic groups (Table 2 and Figure 2). However, mALDHs are very different in various AAB strains. For instance, in term of the subunit composition, mALDHs from both *A. aceti* and *K. europaeus* are heterotrimers (I-II-III), while the mALDH from *A. peroxydans* (currently *A.pasteurianus*) is a heterodimer (I-II; Gómez-Manzo et al., 2010). In term of the prosthetic groups, the mALDH ones from *K. europaeus* include heme *b*, [2Fe-2S] cluster and MCD, while the mALDH ones from *Ga. diazotrophicus* include PQQ, heme *b* and *c* (Thurner et al., 1997; Gómez-Manzo et al., 2010).

As with mADH, mALDH possesses a poor substrate specificity, which can oxidize acetaldehyde, 1-propionaldehyde, 1-butyraldehyde, isobutyraldehyde, glutaraldehyde, and other major short-chain aldehydes except for formaldehyde (Toyama et al., 2007).

#### Flavoprotein–Cytochrome Complex

The flavoprotein-cytochrome complex mDH of AAB mainly includes membrane-binding gluconate dehydrogenase (mGADH) [EC 1.1.99.3], 2-keto-d-gluconate dehydrogenase (m2-KGDH) [EC 1.1.99.4], d-fructose dehydrogenase (mFDH) [EC 1.1.99.11], and d-sorbitol dehydrogenase (mSLDH) [EC 1.1.99.21] (Toyama et al., 2005; Kawai et al., 2013; Kataoka et al., 2015). These mDHs generally consist of a large subunit (I) with FAD as a coenzyme, a medium-size subunit (II) containing three heme *c* prosthetic groups, and a small subunit (III) with unknown function (Table 2 and Figure 2; Toyama et al., 2007).

mGADH, m2-KGDH, mFDH, and mSLDH are all UQ oxidoreductases with high substrate specificity. mGADH is also called GA 2-DH because it can oxidize the C-2 hydroxyl group of GA to produce 2-KGA. mGADH can only oxidize GA, and m2-KGDH can only oxidize 2-KGA to produce 2, 5-DKGA. mFDH can just oxidize fructose to produce 5-KF, which can be used as a biometric recognition molecule of the fructose biosensor. mSLDH can oxidize d-sorbitol to l-sorbose, and also weakly oxidize d-mannitol, whereas pentitol and erythritol are not oxidized by mSLDH (Shinagawa et al., 1982, 1984).

#### Membrane-Binding Quinoprotein

The membrane-binding quinoprotein AAB mDHs mainly include glucose dehydrogenase (mGDH) [EC 1.1.99.17], glycerol dehydrogenase (mGLDH) [EC 1.1.1.6], quinic acid dehydrogenase (mQDH) [EC 1.1.99.25], and inositol dehydrogenase (mIDH) [EC 1.1.1.18], which are composed of an N-terminal transmembrane domain and a C-terminal catalytic domain, including a large subunit (I) and a medium-size subunit (II). The coenzyme of the large subunit is PQQ, which plays a catalytic role, while the medium-size subunit does not contain any coenzyme and is only responsible for binding to the cell membrane (Table 2 and Figure 2).

mGDH is a d-glucose ubiquinone oxidoreductase, which can oxidize the C-1 hydroxyl group of d-glucopyranose to gluconic acid-δ-lactone (GAL; Ameyama et al., 1981), and GAL can be transformed into GA spontaneously or by one of glucolactonases on the cell membrane. mGDH has been developed as a glucose sensor because of its high substrate specificity, which can oxidize only glucose but not hexose and pentose. mGLDH is a glycerol ubiquinone oxidoreductase with the poor substrate specificity, which can oxidize glycerol to dihydroxyacetone, arabitol, sorbitol, mannitol, erytritol, ribiol, and other polyols to the corresponding ketones (Sugisawa and Hoshino, 2002), and GA to 5-KGA (Matsushita et al., 2003). Industrially, mGLDH from *Glucobacter* spp. has been used to produce l-sorbate, DHA, erythrose, and 5-KGA (Shinjoh et al., 2002; Sugisawa and Hoshino, 2002; Hoshino et al., 2003). mQDH is a quinic acid ubiquinone oxidoreductase, which can oxidize the C3 hydroxyl group of quinic acid to 3-dehydroquinic acid (3-DQA), then 3-DQA is transformed to shikimic acid and protocatechuic acid successively by mQDH, of which activity is only one fourth of that of quinic acid (Vangnai et al., 2010). mIDH is an inositol ubiquinone oxidoreductase, which can oxidize the C2 hydroxyl group of inositol to 2-keto-inositol (Holscher et al., 2007).

#### Other Membrane-Binding Dehydrogenases (mDH)

Other types of AAB mDHs include membrane-binding sorbose dehydrogenase (mSDH) [EC 1.1.99.12] and sorbosone dehydrogenase (SNDH) [EC 1.1.1.-] (Table 2). mSDH is an l-sorbose ubiquinone oxidoreductase with FAD as a coenzyme, which can oxidize the C1 hydroxyl group of sorbose to l-sorbosone (Sugisawa et al., 1991). mSDH has a high substrate specificity, which can only oxidize l-sorbose, not other sugars and alcohols (Pappenberger and Hohmann, 2013). There are 2 classes of SNDH, the first class of SNDH is a l-sorbosone ubiquinone oxidoreductase with NAD or NADP as a coenzyme, and can oxidize the C1 hydroxyl group of l-sorbosone to produce 2-keto-l-gulonic acid (2-KGLA; Pappenberger and Hohmann, 2013), present in the cytosol (Sugisawa et al., 1991; Shinjoh and Hoshino, 1995); the other class exists on the plasma membrane(mSNDH), are PQQ-dependent enzymes with l-sorbosone oxidation activity (Yakushi et al., 2020). Recently, it has been suggested that the substrate of mSNDH is the hemiacetal of l-sprbitol-1,5-pyranose (an isoform of l-sorbitol), that oxidized to 2-keto-l-gulone-1,5-pyranose(2-KGLL), and 2-KGLL will spontaneously be hydrolyzed into 2-KGLA under neutral pH conditions, but the substrate specificity, dynamics and structure of such mSNDH still need further investigation (Yakushi et al., 2020).

### Terminal Oxidase (TO) in AOF

The TOs involved in the AOF respiratory chain mainly include UOX and HBD-O. Up to now, the TOs from *G. oxydans* and *A. aceti* have been intensively investigated.

#### Terminal Oxidases (TOs) in G. oxydans

The TOs in *G. oxydans* include cytochrome *bo*_3_-UOX and HBD-O (Prust et al., 2005; Miura et al., 2013; Richhardt et al., 2013; Matsushita et al., 2016). When *bo*_3_-UOX is absent, the early growth of *G. oxydans* is severely affected, whereas when HBD-O is absent, the early growth of cells is not affected (Matsushita et al., 1984). Further studies have indicated that at the early growth stage of *G. oxydans*, when the media pH is neutral, *bo*_3_-UOX is the major TO, and with the accumulation of acid and other products, when the media pH decrease to acid, HBD-O can replace *bo*_3_-UOX as the major TO, and act synergistically with cDH in the cytoplasm, leading that *G. oxydans* cells in acidic conditions continue to utilize organic acids and other products to grow (Miura et al., 2013; Richhardt et al., 2013).

#### Terminal Oxidases (TOs) in A. aceti

The TOs in *A. aceti* includes *ba*_3_/*bo*_3_-UOX, HBD-O, and two homologs of HBD-O, cyanide insensitive oxidase (CIO), CIO1, and CIO2, four TOs in total. Among them, *ba*_3_/*bo*_3_-UOX, CIO1 and CIO2 have a low affinity with oxygen, but a high turnover rate (efficiency), whereas HBD-O has a high affinity with oxygen, and can perform aerobic respiration under hypoxic conditions (Cunningham et al., 1997). Compared with HBD-O, CIO1 and CIO2, *ba*_3_/*bo*_3_-UOX have the stronger ability to form transmembrane proton potential and produce ATP, leading that *ba*_3_/*bo*_3_-UOX is the major TO in *A. aceti* (Matsutani et al., 2014).

## Natural Products Yielded by AOF

**Figure 3:**
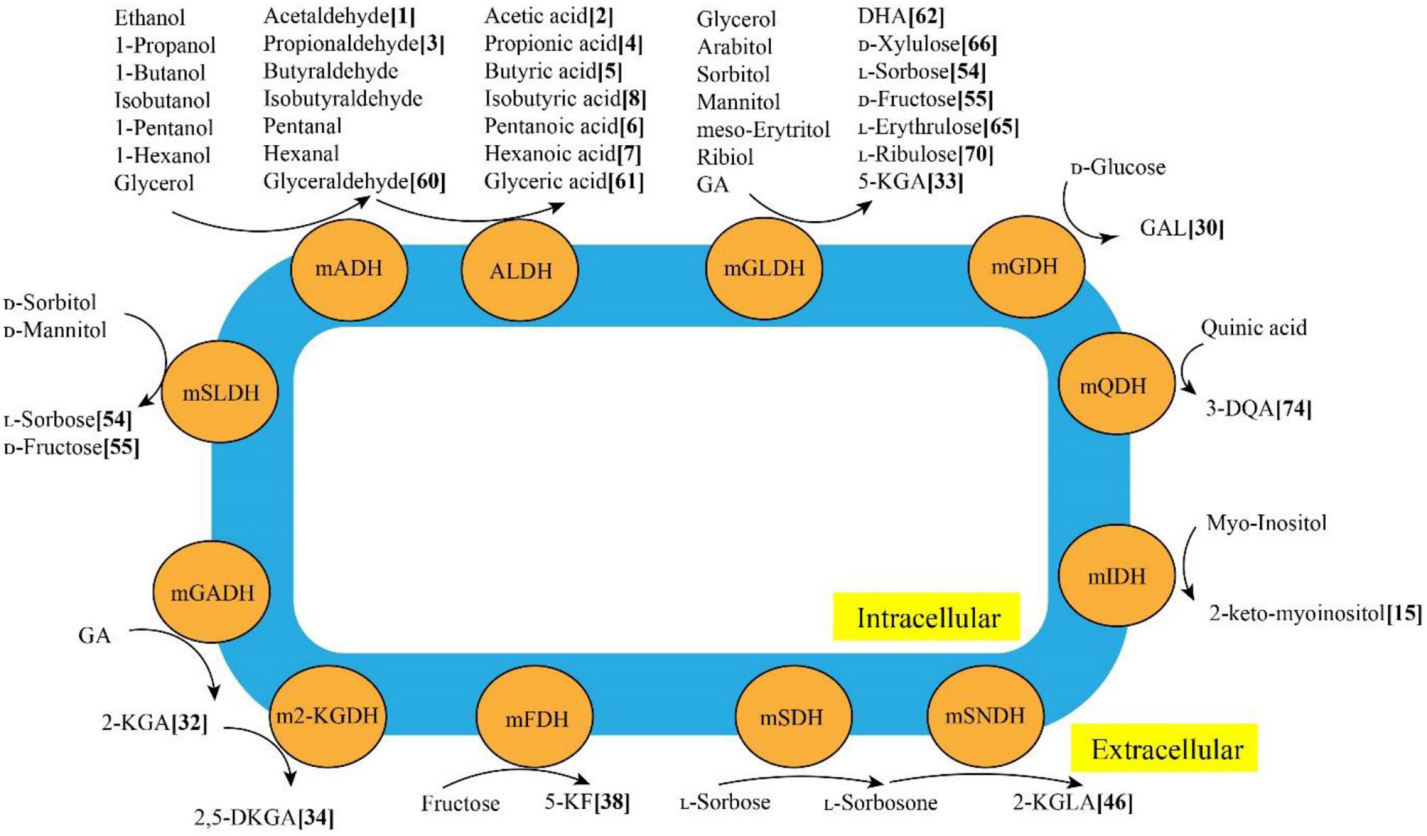
Typical membrane-binding dehydrogenases in acetic acid bacteria and their main AOF products. mADH: membrane-binding alcohol dehydrogenase; mALDH: membrane-binding acetaldehyde dehydrogenase; mGADH: membrane-binding gluconate dehydrogenase; m2-KGDH: membrane-binding 2-keto-D-gluconate dehydrogenase; mFDH: membrane-binding D-fructose dehydrogenase; mSLDH: membrane-binding D-sorbitol dehydrogenase; mGDH: membrane-binding glucose dehydrogenase; mGLDH: membrane-binding glycerol dehydrogenase; mQDH: membrane-binding quinic acid dehydrogenasw; mIDH: membranebinding inositol dehydrogenase; mSDH: membrane-binding sorbose dehydrogenase; mSNDH: membrane-binding sorbone dehydrogenase; GA: gluconic acid; 2-KGA: 2-keto-Dgluconic acid; 2, 5-DKGA: 2, 5-diketo-D-gluconic acid; 5-KF: 5-keto-D-fructose; DHA: dihydroxyacetone; 3-QDA: 3-dehydroquinic acid; 2-KGLA: 2-keto-l-gulonic acid.

Through various mDHs in AOF, AAB can oxidize various alcohols, sugars, sugar alcohols, acids and so on to the corresponding products such as acetic acid, GA, galactonic acid, 2-KGA, DHA, miglitol and so on (Mamlouk and Gullo, 2013), which have been successfully used in foods, cosmetics, medicines and other fields (Gullo et al., 2014; Saichana et al., 2015). The schematic diagram of typical mDHs from AAB strains and their main AOF products is shown in Figure 3. Up to now, 86 AOF products have been reported, and each AOF product is given a bold Arabic numeral (Supplementary Table 2). Based on the numbers in square brackets after each AOF product, the molecular and structural formula of the corresponding AOF compound can be found in Supplementary Table 2.

### AOF Products From Alcohols

AAB can partly oxidize a great number of primary, secondary and diol alcohols to yield the corresponding products, which have been utilized in foods, chemicals, and medicines.

#### Products From Primary Alcohols

The oxidation of ethanol to acetic acid may be the first-known AOF process from the AAB genus *Acetobacter* (Atkinson, 1956). The ethanol oxidation into acetic acid is a typical AOF process, which is divided into two steps (Matsushita et al., 1994). Ethanol is first oxidized to acetaldehyde **[1]** by PQQ-mADH, then converted to acetic acid **[2]** by PQQ-mALDH or MCD-mALDH. Besides the genus *Acetobacter*, the strains from *Komagataeibacter* spp. have very strong abilities to convert ethanol to acetic acid. Moreover, both of them have a high tolerance to ethanol and acetic acid, therefore they are the main species and strains in the vinegar production in the world (Adachi et al., 1978; Kanchanarach et al., 2010).

Except for methanol, mADH and mALDH can incompletely oxidize primary aliphatic normal alcohols with carbon chain length ≤ 6 to corresponding aldehydes and/or acids due to their poor substrate specificities (Adachi et al., 1978; Toyama et al., 2007), resulting in propionaldehyde **[3]** to propionic acid **[4]** from propanol, and butyric acid **[5]**, pentanoic acid **[6]**, hexanoic acid **[7]**, isobutyric acid **[8]**, and isovaleraldehyde **[9]** from butanol, pentanol, hexanol, isobutanol, and isoamyl alcohol, respectively (Švitel and Kutnik, 1995; Švitel and Šturdík, 1995; Molinari et al., 1996; Noyori, 2002).

Moreover, mADH and mALDH can also covert aromatic and other primary alcohols to the respective aldehydes and/or acids including phenylacetaldehyde **[10]**, phenylacetic acid **[11]**, 2-chloropropionic acid **[12]**, (S)-2-phenyl-1-propionic acid **[13]**, 2-methylbutanoic acid **[14]** and 2-keto-myoinositol **[15]** from 2-phenyl-ethanol, 2-chloropropanol, race-2-phenyl-1- propanol, 2-methylbutanol and myo-inositol, respectively (Molinari et al., 1996, 1999; Romano et al., 2002; Gandolfi et al., 2004; Hölscher and Görisch, 2006; Keliang and Dongzhi, 2006). Wei et al. (2016) found that racemic 1-(4-methoxyphenyl) ethanol (race-MOPE) could be oxidized to enantiopure (S)-MOPE **[16]** and 4-methoxyacetophenone **[17]** by mADH from *Acetobacter* sp. CCTCC M209061.

#### Products From Secondary Alcohols and Diols

Švitel and Kutnik (1995) found that *G. oxydans* CCM1783 could oxidize isopropanol and 2-butanol to acetone **[18]** and 2-buranone **[19]**, respectively. Some AAB strains can also stereoselectively oxidize 2-methy-1,3-propanediol to (R)-β-hydroxyisobutyric acid **[20]** which is an important chiral building block in the synthesis of drugs (León et al., 2009).

The diols can be oxidized by some AAB strains, too. For example, 1,3-butandiol, 2,3- butandiol, (2R,3R)-2,3-butandiol, *N*-2-1,4-nonanodiol, 1,2-propanediol, ethanediol (ethylene glycol), racemical-1,2-butanediol, 1,3-propanediol and (R)-1-phenyl-1,2-ethanediol are oxidized to 3-hydroxybutyric acid **[21]** (Romano et al., 2002), (S)-acetoin **[22]** (Romano et al., 2002; Wang et al., 2013; Zhou et al., 2018), diacetyl **[23]**, γ-nonanoic lactone **[24]** (Romano et al., 2002), (R)-2-hydroxy-propionic acid (d-(-)-lactic acid) **[25]** (Su et al., 2004), glycolic acid **[26]** (Wei et al., 2009), (R)-2-hydroxybutyric acid **[27]** (Gao et al., 2012), 3-hydroxypropionic acid **[28]** (Dishisha et al., 2015; Zhu et al., 2018), and (R)-mandelic acid **[29]** (Li et al., 2014), respectively.

### Products From Sugars and Disaccharides *via* AOF

Through AOF, AAB can partially oxidize various sugars and disaccharides like glucose, fructose, arabinose, ribose, xylose, sorbose, lactose, isomaltose, gentiobiose, and melibiose to the corresponding products.

#### Products From Glucose

During AOF, glucose is first converted into gluconic acid-δ-lactone **[30]** (Shinagawa et al., 2009), then changed into GA **[31]** spontaneously or by membrane-binding gluconic acid-δ- lactonase (Shinagawa et al., 2009). GA can further be oxidized to 2-KGA **[32]** by GA dehydrogenase, or to 5-KGA **[33]** by PQQ dependent glycerol dehydrogenase (PQQ-GLDH); 2-KGA can be catalyzed to 2,5-DKGA **[34]** by FAD-dependent 2-keto-d-gluconic acid dehydrogenase (FAD-GADH; Matsushita et al., 2003; Shinagawa et al., 2009). 2,5-DKGA can be transferred to 4-keto-d-arabinose **[35]**, which is further catalyzed to 4-keto-d-arabonate **[36]** by 4-keto-d-aldopentose-1-dehydrogenase (Adachi et al., 2011a). 2,5-DKGA can be decarboxylated to form d-lyxuronic acid **[37]**, too (Kondô and Ameyama, 1958).

Glucose oxidative fermentation of AAB is not only closely related to glucose concentration and reaction pH (Qazi et al., 1991) but also to the sugars of the culture medium. When glucose in the medium is depleted, small amounts of 2-KGA and 5-KGA secreted in the medium can be transported to AAB cells by transporters and then reduced to GA by 2-KGA reductase (2-KGAR) or 5-KGA reductase (5-KGAR) in the cytoplasm, and utilized by AAB through the pentose phosphate pathway (PPP), allowing AAB to reproduce again and present a secondary growth curve (Saichana et al., 2015). The kinds of products obtained from glucose oxidation by AAB are also affected by pH, for example, when the pH of the culture medium is at 3.4-4.0, AAB oxidized glucose produces only 5-KGA. Therefore, when producing GA, 2-KGA, and 5-KGA using AAB species, to prevent them from being further consumed by AAB utilization, culture conditions with high glucose concentration, low pH and high O_2_ must be applied and fermentation terminated before the appearance of the secondary growth curve (Mamlouk and Gullo, 2013).

#### Products From Fructose, Arabinose, Ribose, Xylose, Sorbose, and Galactose

d-fructose can be oxidized to 5-keto-d-fructose **[38]** *via* mFDH and d-pentonate-4- dehydrogenase (P-4-DH) from AAB cells (Adachi et al., 2011b; Ano et al., 2017). mFDH possesses a high substrate specificity, whereas P-4-DH does not, which can oxidize d-psicose to 5-keto-d-psicose **[39]** (Ano et al., 2017**)**, and shows mGLDH activity, too. In addition, fructose turns to glucosone **[40]** *via* a series of oxidized directly by *G. roseus* (Takahashi and Asai, 1932; Ikeda, 1955).

AAB strains can also transform d-arabinose to 4-keto-d-arabinose **[35]** and 4-keto-d-arabonate **[36]** (Adachi et al., 2010); d-ribose to 4-keto-d-ribose **[41]** and 4-keto-d-ribonate **[42]** (Adachi et al., 2011a); xylose to xylonic acid **[43]** (Buchert et al., 1988; Hahn et al., 2020), d-xylulose to xylitol **[44]** (Qi et al., 2016), sorbose to sorbitol **[45]** and 2-keto-d-gulonic acid **[46]** (Sugisawa et al., 1991; Adachi et al., 1999a), and d-galactose to d-galactonic acid **[47]** (Švitel and Šturdik, 1994). Adachi et al. (2013) also found that AAB cells could use 2-deoxy-d-ribose as a substrate to produce 2-deoxy-4-keto-d-ribose **[48]** and 2-deoxy-4-keto-d-ribonate **[49]**, which are under the action of membrane-binding d-aldopentose 4-dehydrogenase and 4-keto-d-1-dehydrogenase, respectively (Adachi et al., 2013).

#### Products From Disaccharides

The strain *A. orientalis* KYG 22 can oxidize lactose to lactose acid **[50]** with the aid of d-glucose by mGDH (Kiryu et al., 2014). mGDH from some strains of *Gluconobacter, Ga. hansenii* NBRC 14816 and *K. medellinensis* NBRC 3288 can also oxidize isomaltose, gentiobiose and melibiose to isomaltobionic acid **[51]**, gentiobionic acid, **[52]**, and melibionic acid **[53]**, respectively (Kiryu et al., 2020).

### Products From Sugar Alcohols Through AOF

The sugar alcohols such as d-sorbitol, glycerol, d-mannitol, d-arabinol, d-erythritol and d-ribitol are transferred by AOF into l-sorbose, DHA, l-erythrose, d-xylulose, d-fructose, and l-ribulose, respectively (Cummins et al., 1957; Sugisawa and Hoshino, 2002; Prust et al., 2005; Adachi et al., 2007; Mamlouk and Gullo, 2013).

#### Products From Sorbitol

Sorbitol may be sequentially oxidized by two mDHs, sorbitol dehydrogenase and sorbose dehydrogenase from AAB cells, to l-sorbose **[54]** and d-fructose **[55]** (Cummins et al., 1957; Sato et al., 1967). Then sorbose is changed to 5-keto-d-fructose **[56]**, which will be converted into three γ-pyrone compounds including kojic acid **[57]**, 3-oxykojic acid, **[58]**, and 5-oxymaltol **[59]** (Sato et al., 1967). In addition, l-sorbosone can enter AAB cells, then transformed to 2-keto-l-gulonic acid **[46]**, which is secreted extracellularly as an intermediate of vitamin C synthesis (Sugisawa and Hoshino, 2002; Matsushita et al., 2003; Adachi et al., 2007).

#### Products From Glycerol

The *G. frateurii* NBRC3262 strain can sequentially convert glycerol to glyceraldehyde **[60]** to d-glyceric acid **[61]** by mADH (Habe et al., 2009). Some *G. oxydans* strains can oxidize glycerol to DHA **[62]** *via* mGLDH (Habe et al., 2009; Hu et al., 2010). DHA is widely used in cosmetics as an active sunscreen ingredient, as well as utilized in weight loss, antioxidant, vitiligo treatment, and so on (Levy, 1992). Some *Acetobacter* strains can convert racemic glycidol to glycidic acid **[63]** (Geerlof et al., 1994; Švitel and Kutnik, 1995).

#### Products From Other Sugar Alcohols

*A. suboxydans* strains can make l-fucitol convert to l-duco-4-ketose **[64]** (Richtmyer et al., 1950). Meso-erythritol is changed to l-erythrulose **[65]** by AAB cells (Richtmyer et al., 1950). d-arabitol (the 2-epimer of eylitol) was firstly oxidized to d-xylulose **[66]** by d-arabitol dehydrogenase (AraDH), then reduced to xylitol **[67]** by NAD-dependent xylitol dehydrogenase (Suzuki et al., 2002; Liu et al., 2019). Xylitol, a natural pentahydroxy sugar alcohol, has a sweet compound similar to sucrose and serves as a substitute for natural sweeteners. It also plays a role in preventing tooth decay (Suzuki et al., 2002). *G. suboxydans* can produce d-fructose **[55]** from d-mannitol (Adachi et al., 1999b). *Ac. oxydans* and could oxidize allitol to l-allulose (l-ribo-hexulose or l-psicose) **[68]** (Carr et al., 1968; Takeshita et al., 1996), which is rare and not natural ketohexose. AAB cells can also oxidize ribitol to l-ribulose **[60]** by membrane-binding NAD(P) independent ribitol dehydrogenase (Adachi et al., 2001). Recently, Xu et al. (2021) reported that polyol galactitol can be oxidized to two rare sugars, d-tagatose **[70]** and l-xylo-3-hexulose **[71]** by PQQ-dependent l-arabinitol 4-dehydrogenase (PQQ-LAD) from *Acetobacter* sp. and *Gluconobacter* sp. strains.

### Products Converted From Organic Acids

#### Products From Quinate

Whiting and Coggins (1967) firstly reported that quinate oxidase from *Ac. oxydans*, quinate-cytochrome 555 oxidoreductase, could oxidize d-dihydroshikimic acid into 3,4- dihydroxy-5-oxocyclohexane-1-carboxylic acid **[72]**. Quinate is oxidized to 3-dehydroquinate (DQA) **[73]** by NAD(P) independent quinate dehydrogenase (QDH, EC 1.1.99.25), then to 3-dehydroshikimate (DSA) **[74]** *via* DQA dehydratase (EC. 4.2.1.10), and DSA can be converted to protocatechuate **[75]** by DSA dehydratase (Adachi et al., 2006).

#### Products From Other Organic Acids

Benziman and Perez (1965) proved that malate was converted to oxaloacetate **[76]** by a FAD-protein from *A. xylinum* (currently *K. xylinus*). Dosoretz et al. (1992) reported that the pure cultures of *A. pasteurianus, G. cerinus* and *G. oxydans* could oxidize calcium magnesium (Ca-Mg) lactate to Ca-Mg acetate (CMA) **[77]**, which is considered to be a potentially noncorrosive and biodegradable deicing chemical. In 2015, Sato et al. discovered that some strains of *Acetobacter* spp. could partially oxidize l/d-lactate into pyruvate **[78]** by lactate dehydrogenase (Sato et al., 2015).

### Products Converted From Other Substrates *via* AOF

#### Products From Hexosamine

In 1960, Takahashi and Kayamori discovered that *A. melanogenum* (currently *G. oxydans*) could transform glucosamine into glucosaminic acid **[79]**, which is used as a commercial chemical in daily life (Takahashi and Kayamori, 1960). In 2004, Moonmangmee et al. found that d-mannosamine and d-galactosamine were changed into d-mannosaminate **[80]** and d-galatosaminate **[81]** by *Gluconobacter* sp. IFO 3264, respectively (Moonmangmee et al., 2004).

#### Products From Furfural

Zhou et al. (2017) prove that *G. oxydans* ATCC 621H could change furfural or furfuryl alcohol into 2-furoic acid **[82]**, which is the raw material to produce many furoate esters and its derivatives widely used in the synthesis of pharmaceutical, agricultural and industrial chemicals (Zhou et al., 2017). Sayed et al. (2019) found that the resting cells of *G. oxydans* DSM 50049 were capable of highly selective production of 5-hydroxymethyl-2- furan-carboxylic acid **[83]**, which is used as a monomer of a variety of polymers with antibacterial and antitumor activities (Sayed et al., 2019).

#### Products From Others

Sugisawa et al. (2005) first found that *G. oxydans* DSM 4025 could produce l-ascorbic acid **[84]** from l-gulono-γ-lactone with l-gulonate-γ-lactone dehydrogenase. Landis et al. (2002) prove that the strains of *G. oxydans* could also selectively region oxidize the *N*- butylglucamine into 6-deoxy-6-butylaminosorbose **[85]** (Landis et al., 2002). Some AAB strains can yield 6-(2-hydroxy-ethyl) amino-6-deoxy-α-l-sorbofuranose **[86]** using *N*-2-hydroxyethyl glucamine as substrate, which is an important precursor of miglitol, a α-glucosidase inhibitor, which was approved by the Food and Drug Administration of United States for the treatment of type II diabetes in December 1996 (Keliang and Dongzhi, 2006).

## Molecular Biological Methods for AOF

In recent decades, a lot of molecular biology techniques have been applied to investigate AOF, especially its mDHs. Hereinafter, we just make a brief introduction about the application of molecular biology technologies in AOF, especially the establishment of a marker-less gene deletion system, for more detailed information, please read our recent review article (Yang et al., 2022).

### Establishment of Marker-Less Gene Deletion System and Its Application in AOF

To investigate the function of mDH's genes from AAB, gene deletion is doubtlessly a direct and powerful tool. In this context, using a marker-less strategy is preferable to other molecular biology methods due to the following reasons (Peters et al., 2013): (1) the number of available markers such as antibiotic-resistant gene markers is so limited that the construction of the multi-gene knockout strain is sometimes impossible with marker genes; (2) as about 50% of genes in prokaryotes are located in the operons, where genes are co-transcribed into a single polycistronic mRNA (Osbourn and Field, 2009), the insertion of marker genes into the operon may exert polar effects and suppress the expression of downstream genes in the same operon.

Marker-less gene deletion techniques are often carried out in a two-step procedure using a plasmid vector carrying an antibiotic resistance selection marker, a counter selection marker, as well as the fused flanking fragments of the target gene (Gao et al., 2006). In the first step, the non-replicating plasmid vector is introduced into the host cell. Subsequently, the clones with the vector integrated at either the upstream or downstream site of the target gene by the homologous recombination are screened by antibiotic resistance and will be used for the second homologous recombination, in which the clones without the vector will be obtained through the recombination of the other homologous flanking region and the counter selection marker. The second homologous recombination will yield either the wild type strain or the desired gene deletion mutant with the theoretical ratio of 1:1 (Yang et al., 2022).

Peters et al. (2013) reported such a gene deletion system for AAB. The system involves a kanamycin resistance gene to select for AAB cells harboring the vector after its integration at the target site of the genome, together with gene *upp*, encoding uracil phosphoribosyl- transferase for the subsequent counter-selection to lose the vector from the genome, which could result in the desired deletion mutant without any marker sequence. In this case, uracil phosphoribosyltransferase converts the counter selection agent 5-fluorouracil (FU) to toxic 5-fluorouridinemonophosphate (F-UMP) to kill the wild type. However, most AAB species, including *G. oxydans*, possess the *upp* gene in their genomes. Therefore, deletion of the *upp* gene is required prior to application of this deletion method.

In order to avoid the deletion of *upp* before knocking out other genes, an improved method was established by Kostner et al. (2013) using the *codA* gene from *Echerichia coli* as the counter selection marker. This gene codes for cytosine deaminase, converting the nontoxic counters election agent 5-fluorocytosine (FC) to FU, which is subsequently converted to toxic F-UMP by uracil phosphoribosyltransferase encoded by gene *upp*. In addition, they found that the co-expression of *codA* with *codB*, coding for cytosine permease that facilitate the uptake of FC, can significantly increase the efficiency of the deletion method. Thus, *codB* was introduced to the deletion vector and also became a part of counter selection marker together with *codA* in this newly developed system.

Using the *upp* marker-based counter selection approach, Peters et al. (2013) knocked out all mDH-coding genes in *G. oxydans* 621H in a sequential manner, creating a series of mutants lacking one or more mDH (s), including *G. oxydans* BP.9 without all mDH genes and *G. oxydans* BP.8 only with the gene of a polyol dehydrogenase. Subsequently, by adopting a whole-cell 2,6-dichlorophenolindophenol (DCPIP) activity assay, the substrate specificities of the mDHs were clarified. Mientus et al. (2017) analyzed the substrate spectra of the mDHs of *G. oxydans* by applying a shuttle vector to express each individual enzyme in *G. oxydans* BP.9, and the substrate spectra of every one of the mDHs were defined by the whole-cell DCPIP assay. In addition, Peters et al. (2013) used *G. oxydans* BP.9 to express genes encoding mDHs from the metagenome of a mother of vinegar sample including many non-culturable AAB and other microorganisms. In 2019, Burger et al. applied *G. oxydans* BP.8 to investigate and optimize the production of l-erythrulose (Burger et al., 2019).

### Molecular Biology Studies Regarding AOF Other Than Marker-Less Deletion

Besides the gene marker-less deletion method, a number of other molecular biological technologies have also been applied for AOF research. For example, PQQ-GLDH was once conferred different names owning to the diversity of its substrates, including d-gluconate, d-arabitol, and d-sorbitol (Shinagawa et al., 1999; Adachi et al., 2001; Sugisawa and Hoshino, 2002). It was not until the gene disruption experiments that people finally realized that these enzymes are actually the same (Miyazaki et al., 2002; Shinjoh et al., 2002). Later, the name PQQ-GLDH was confirmed by Matsushita et al. (2003).

The molecular techniques are also exploited with the aim of promoting AOF production. A typical example is the oxidation of d-glucose, which is the most common substrate for *G. oxydans* and can be transferred into a variety of different products, such as GA, 5-KGA, 2-KGA, and 2, 5-DKGA (Saichana et al., 2015). Therefore, in terms of GA production, further oxidation of GA to ketogluconate is unexpected, suggesting that promotion of GA could be achieved by suppressing the mDHs which can further oxidize GA (La China et al., 2018). On the other hand, an increase in the production of GA, 2-KGA, and 5-KGA could be achieved by over-expression of genes encoding FAD-dependent d-gluconate dehydrogenase (Shi et al., 2014), PQQ-GDH and PQQ-GLDH (Merfort et al., 2006), respectively. In addition, either enhancing the abundance of mRNA transcribed from PQQ-GLDH-coding genes through adding an A/T tail (Xu et al., 2014) or over-expression of genes responsible for PQQ biosynthesis (Wang et al., 2016) can lead to the improved production of l-sorbose, which could be used as the substrate for producing 2-keto-l-gulonic acid, a direct precursor of vitamin C (La China et al., 2018). Furthermore, Gao et al. (2014) heterologously expressed a different combination of genes encoding five l-sorbose mDHs and two l-sorbosone mDHs from *Ketogulonicigenium vulgare* in *G. oxydans* WSH-003, and screened the best recombinant strain *G. oxydans*/pGUC-*k*0203-GS-*k*0095 with the highest yield, achieving one-step production of 2-keto-l-gulonic acid from d-sorbitol, which was produced *via* a two-step process in the past.

## Conclusion and Discussion

AAB is a large group of Gram-negative, strictly aerobic bacteria, some of which have the great ability to yield a number of products with commercial or potential commercial values by their unique oxidative fermentation (AOF). In this review, we first summarize the AAB classification progress, then systematically describe and classify AOF products and the relative enzymes. The application of molecular biology technologies in AOF research is also briefly introduced.

Although many research works have been carried out and remarkable progress has been made on AOF, some AOF products such as acetic acid (vinegar), bacterial cellulose, DHA and so on (La China et al., 2018), have been industrialized, there are still a lot of issues about AOF which need to be further investigated. For example, although we are pleased to witness the promising progress achieved in the research on the functions of AAB mDHs, as well as in the AOF application, 21 'orphan' mDHs with unknown substrate spectra in *G. oxydans* are still unexplored (Adachi and Yakushi, 2016), and few crystal structures of AAB mDHs have been published (Qin et al., 2022), which greatly hinders the understanding of substrate space- and enantio- specificities of AAB mDHs. Moreover, the relevant modern technologies, especially modern molecular biotechnology including omics, systems biology, combinatorial biology, synthetic biology, and metabolic engineering, should be utilized for constructing relevant engineering strains for efficient production of the target AOF products.

## Author Contributions

YH contribute to the data collection, collation, and writing of the first draft. ZX and HZ responsible for the data collection and collation. WL and HT provide brief article ideas and language modifications. FC propose the overall idea for this article, reviewed and revised the first draft, and provided the corresponding financial support. All authors contributed to the article and approved the submitted version.

## Funding

This work was supported by the Fundamental Research Funds for the Central Universities (No. 2662019PY015), the Major Special Projects of Technological Innovation of Hubei Province, China (No. 2018ABA075), the Major Science and Technology Project in Zhenjiang City, Jiangsu Province, China (No. ZD2019001), Programs of the International S&T Cooperation, Ministry of Science and Technology, China (No. 2014DFG32380), and China Scholarship Council (202006760070).

## Conflict of Interest

The authors declare that the research was conducted in the absence of any commercial or financial relationships that could be construed as a potential conflict of interest.

## Publisher's Note

All claims expressed in this article are solely those of the authors and do not necessarily represent those of their affiliated organizations, or those of the publisher, the editors and the reviewers. Any product that may be evaluated in this article, or claim that may be made by its manufacturer, is not guaranteed or endorsed by the publisher.
