# Supplementary material for: Oxidative Fermentation of Acetic Acid Bacteria and Its Products
Source: Front Microbiol. 2022 May 24;13:879246. doi: 10.3389/fmicb.2022.879246 (PMC9171043; doi:10.3389/fmicb.2022.879246)
Supplement: Supplementary file 1 [file Data_Sheet_1.docx]

**Suppl. Table 1 The name lists of genera, species and typical strains of acetic acid bacteria**

| **Genera** | **Species and their typical strains** | |
| --- | --- | --- |
| *Acetobacter* ( *A.*） | *A. aceti* | IFO 14818 |
|  | *A. cerevisiae* | LMG 1625 |
|  | *A. cibinongensis* | IFO 16605 |
|  | *A. conturbans* | LMG 1627 |
|  | *A. estunensis* | IFO13751 |
|  | *A. fabarum* | LMG 24244 |
|  | *A. fallax* | LMG 1636 |
|  | *A. farinalis* | NBRC 107750 |
|  | *A. garciniae* | LMG 32243T |
|  | *A. ghanensis* | LMG 23848 |
|  | *A. indonesiensis* | IFO 16471 |
|  | *A. lovaniensis* | IFO 13753 |
|  | *A. lambici* | LMG 27439 |
|  | *A. malorum* | LMG 1746 |
|  | *A. musti* | DSM 23824 |
|  | *A. nitrogenifigens* | LMG 23498 |
|  | *A. oeni* | LMG 21952 |
|  | *A. okinawensis* | LMG 26457 |
|  | *A. orientalis* | IFO 16606 |
|  | *A. orleanensis* | IFO 13752 |
|  | *A. papayae* | LMG 26456 |
|  | *A. pasteurianus* | IFO 13755 |
|  | *A. peroxydans* | JCM 25077 |
|  | *A. persicus* | LMG 26458 |
|  | *A. pomorum* | DSM 11825 |
|  | *A. senegalensis* | LMG 23690 |
|  | *A. suratthanensis* | NBRC 111399 |
|  | *A. syzygii* | IFO 16604 |
|  | *A. tropicalis* | IFO 16470 |
|  | *A. ascendens* | LMD 51.1T |
|  | *A. oryzoeni* | B6T |
|  | *A. oryzifermentans* | JCM 31096 |
|  | *A. sicerae* | LMG 1531 |
|  | *A. thailandicus* | NBRC 103583 |
| *Acidomonas* (*Ac*.) | *Ac. methanolica* | IMET 10945 |
| *Ameyamaea* (*Am*.） | *Am. chiangmaiensis* | NBRC 103196 |
| *Asaia* (*As.*) | *As. astilbes* | DSM 23030 |
|  | *As. bogorensis* | JCM 10569 |
|  | *As. krungthepensis* | NBRC 100057 |
|  | *As. lannaensis* | NBRC 102526 |
|  | *As. platycodi* | DSM 23029 |
|  | *As. prunellae* | JCM 25354 |
|  | *As. siamensis* | IFO 16457 |
|  | *As. spathodeae* | NBRC 105894 |
| *Bombella* (*B*.) | *B. intestini* | DSM 28636 |
|  | *B. apis* | JCM 31623 |
|  | *B. favorum* | CECT 30114 |
|  | *B. mellum* | CECT 30113 |
| *Commensalibacter* (*C.*) | *C. intestini* | JCM 15511 |
| *Endobacter* (*E.*) | *E. medicaginis* | LMG 26838 |
|  | *E. cereale* | RZME 27 |
| *Gluconacetobacter* (*Ga.*) | *Ga. asukensis* | JCM 17772 |
|  | *Ga. aggeris* | JCM 19092 |
|  | *Ga. azotocaptans* | ATCC 700988 |
|  | *Ga. diazotrophicus* | LMG 7603 |
|  | *Ga. dulcium* | LMG 1728 |
|  | *Ga. entanii* | DSM 13536 |
|  | *Ga. johannae* | ATCC 700987 |
|  | *Ga. liquefaciens* | IFO 12388 |
|  | *Ga. persimmonis* | KCTC 10175BP |
|  | *Ga. sacchari* | DSM 12717 |
|  | *Ga. takamatsuzukensis* | JCM 19094 |
|  | *Ga. tumulicola* | JCM 17774 |
|  | *Ga. tumulisoli* | JCM 19097 |
| *Gluconobacter* (*G*.) | *G. albidus* | NBRC 3250 |
|  | *G. aidae* | NBRC 103576T |
|  | *G. cadivus* | LMG 1744 |
|  | *G. cerinus* | IFO 3267 |
|  | *G. cerevisiae* | LMG 27748 |
|  | *G. frateurii* | IFO 3264 |
|  | *G. japonicus* | NBRC 3271 |
|  | *G. kanchanaburiensis* | NBRC 103587 |
|  | *G. kondonii* | IFO 3266 |
|  | *G. oxydans* | NBRC 14819 |
|  | *G. roseus* | NBRC 3990 |
|  | *G. potus* | LMG 1764 |
|  | *G. roseus* | IFO 3990 |
|  | *G. sphaericus* | NBRC 2467 |
|  | *G. thailandicus* | NBRC 100600 |
|  | *G. vitians* | LMG 31484 |
|  | *G. wancherniae* | NBRC 103581 |
|  | *G. morbifer* | G 707 |
| *Granulibacter* (*Gr.*) | *Gr. bethesdensis* | DSM 17861 |
| *Komagataeibacter* (*K*.) | *K. europaeus* | DSM 6160 |
|  | *K. hansenii* | NBRC 14820 |
|  | *K. intermedius* | DSM 11804 |
|  | *K. kombuchae* | LMG 23726 |
|  | *K. nataicola* | LMG 1536 |
|  | *K. oboediens* | DSM 11826 |
|  | *K. rhaeticus* | LMG 22126 |
|  | *K. saccharivorans* | LMG 1582 |
|  | *K. swingsii* | LMG 22125 |
|  | *K. sucrofermentans* | LMG 18788 |
|  | *K. xylinus* | NBRC 15237 |
|  | *K. kakiaceti* | JCM 25156 |
|  | *K. medellinensis* | LMG 1693 |
|  | *K. maltaceti* | NBRC 14815 |
|  | *K. melaceti* | LMG 31303 |
|  | *K. cocois* | WE7T |
|  | *K. pomaceti* | LMG 30150(T) |
|  | *K. diospyri* | MSKU 9(T) |
| *Kozakia* (*Ka*.) | *Ka. baliensis* | JCM11301 |
| *Neoasaia* (*N*.) | *N. chiangmaiensis* | NBRC 101099 |
| *Neokomagataea* (*Ne*.) | *Ne. thailandica* | NBRC 106555 |
|  | *Ne. tanensis* | NBRC 106556 |
| *Nguyenibacter* (*Ng*.) | *Ng. vanlangensis* | NBRC 109046 |
| *Saccharibacter* (*S*.) | *S. floricola* | JCM 12116 |
| *Swaminathania* (*Sa*.) | *Sa. salitolerans* | LMG 21291 |
| *Swingsia* (*Si*.) | *Si. samuiensis* | NBRC 107927 |
| *Tanticharoenia* (*T*.) | *T. sakaeratensis* | NBRC 103193 |
|  | *T. aidae* | NBRC 110637 |
| Total 19 genera | 110 species | |

**Suppl. Table 2 AAB oxidative fermentation (AOF) products**

|  | **No.** | **Substrates** | **Products** | **Structures** | **Functions** | **References** |
| --- | --- | --- | --- | --- | --- | --- |
| AOF products from alcohols | 1 | Ethanol | Acetaldehyde |  | Intermediate production of ethanol fermentation | (Adachi et al., 1978) |
|  | 2 | Ethanol | Acetate |  | Vinegar | (Adachi et al., 1978) |
|  | 3 | Propanol | Propionaldehyde |  | Fine chemical raw material | (Adachi et al., 1978) |
|  | 4 | Propanol | Propionic acid |  | Fine chemical raw material | (Švitel and Kutnik, 1995) |
|  | 5 | Butanol | Butyric acid |  | Herbicide, insecticide, flavoring agent etc. | (Adachi et al., 1978) |
|  | 6 | Pentanol | Pentanoic acid |  | Flavors, medicine lubricants and other industries | (Adachi et al., 1978) |
|  | 7 | Hexanol | Hexanoic acid |  | Food flavoring, pharmaceutical industry | (Adachi et al., 1978) |
|  | 8 | Isobutanol | Isobutyric acid |  | Organic synthesis of raw materials, advanced solvents | (Švitel and Kutnik, 1995) |
|  | 9 | Isoamy alcohol | Isovaleraldehyde |  | Aroma compound | (Molinari et al., 1996) |
|  | 10 | 2-Phenyl-1-ethanol | Phenylacetaldehyde |  | Aroma compound | (Molinari et al., 1999) |
|  | 11 | 2-Phenyl-1-ethanol | Phenylacetic acid |  | Aromatic compound | (Geerlof et al., 1994) |
|  | 12 | 2-Chloropropanol | 2-Chloropropionic acid |  | Key intermediates in the synthesis of chiral aromatic oxypropionic acid pesticides | (Romano et al., 2002) |
|  | 13 | 2-Phenyl-1-propanol | 2-Phenylpropionic acid |  | Nonsteroidal anti-inflammatory drugs | (Romano et al., 2002) |
|  | 14 | 2-Methylbutanol | 2-Methylbutanoic acid |  | Aromatic compound | (Keliang and Dongzhi, 2006) |
|  | 15 | Myo-inositol | 2-Keto-myoinositol |  | -- | (Holscher et al., 2007) |
|  | 16 | Rac-1-(4-methoxyphenyl)-ethanol | (S)-1-(4-methoxyphenyl)-ethanol |  | Synthesis of cycloalkyl indoles | (Wei et al., 2016) |
|  | 17 | Rac-1-(4-methoxyphenyl)-ethanol | 4-Methoxyacetophenone |  | Intermediate in fine and specialty chemicals and pharmaceuticals | (Wei et al., 2016) |
|  | 18 | Isopropanol | Acetone |  | Synthesis of methyl isobutyl ketone Flavoring substance, high value | (Gandolfi et al., 2004) |
|  | 19 | 2-Butanol | 2-Butanone |  | Chemical raw material. Use as pains, coatings, medicine, etc. | (Švitel and Kutnik, 1995) |
|  | 20 | 2-Methyl-1, 3-propanedi | (R)-β-Hydroxyisobutyric |  | Important chiral building blocks for drugs | (León et al., 2009) |
|  | 21 | 1, 3-Butandiol | 3-Hydroxybutyric acid |  | Separation of optical isomers, drug intermediates | (Romano et al., 2002) |
|  | 22 | Meso-2,3-Butandiol | (S)-Acetoin |  | - | (Romano et al., 2002 ; Wang et al., 2013) |
|  | 23 | (R, R)-2, 3- butanediol | Diacetyl |  | Synthetic spices | (Romano et al., 2002) |
|  | 24 | N-2- 1,4-Nonandiol | γ-Nonanoic lactone |  | Food spice, high-grade cosmetics used in the fragrance of lactone fragrance | (Romano et al., 2002) |
|  | 25 | 1, 2-Propanediol | (R)-2-hydroxy propionic acid |  | Pesticide, medicine and chemical industry | (Su et al., 2004) |
|  | 26 | Ethylene glycol | Glycolic acid |  | Fine chemical, used in metal cleaning, textiles, leather processing | (Wei et al., 2009) |
|  | 27 | Racemic 1, 2- butanediol | (R)-2-Hydroxybutyric acid |  | Important chiral building block for synthesis of drugs | (Gao et al., 2012) |
|  | 28 | 1, 3-Propanediol | 3-Hydroxypropionic acid |  | Crosslinking agents, lubricants, general purpose compounds for organic synthesis | (Dishisha et al., 2015) |
|  | 29 | (R)-1-Phenyl-1, 2- ethanediol | (R)-Mandelic acid |  | Raw material for pharmaceutical intermediates | (Li et al., 2014) |
| AOF products from sugars | 30 | ᴅ-Glucose | ᴅ-Gluconic acid-δ-lactone |  | Bean protein coagulant | (Shinagawa et al., 2009) |
|  | 31 | ᴅ-Glucose | ᴅ-Gluconic acid |  | Animal fodder | (Shinagawa et al., 2009) |
|  | 32 | ᴅ-Glucose | 2-Keto-ᴅ-Gluconic acid |  | Food additive, cement plasticizer, detergent | (Shinagawa et al., 2009 ; Matsushita et al., 2003) |
|  | 33 | ᴅ-Glucose | 5-Keto-ᴅ-Gluconic acid |  | For ascorbic acid and ʟ-(+)-tartaric acid production | (Shinagawa et al., 2009 ; Matsushita et al., 2003) |
|  | 34 | ᴅ-Glucose | 2,5-Keto-ᴅ-Gluconic acid |  | Important precursor of vitamin C | (Shinagawa et al., 2009 ; Matsushita et al., 2003) |
|  | 35 | ᴅ-Arabinose | 4-Keto-ᴅ-arabinose |  | -- | (Adachi et al., 2011a) |
|  | 36 | ᴅ-Arabinose | 4-Keto-ᴅ-arabonate |  | -- | (Adachi et al., 2011a ; Adachi et al., 2010) |
|  | 37 | ᴅ-Glucose | ᴅ-Lyxuronic acid |  | -- | (Kondô and Ameyama, 1958) |
|  | 38 | ᴅ-Fructose | 5-Keto-ᴅ-fructose |  | -- | (Ano et al., 2017 ; Adachi et al., 2011b) |
|  | 39 | ᴅ-Psicose* | 5-Keto-ᴅ-psicose* |  | -- | (Ano et al., 2017) |
|  | 40 | Fructose | Glucosone |  | For industry use only | (Ikeda, 1955) |
|  | 41 | ᴅ-Ribose | 4-Keto-ᴅ-ribose |  | -- | (Adachi et al., 2011a) |
|  | 42 | ᴅ-Ribose | 4-Keto-ᴅ-ribonate |  | -- | (Adachi et al., 2011a) |
|  | 43 | Xylose | Xylonic acid |  | Platform compounds used in construction, aquaculture, biological control, etc. | (Buchert et al., 1988) |
|  | 44 | ᴅ-Xylulose | Xylitol |  | Anti-caries, used as diabetic food, chemical industry, medicine | (Qi et al., 2016) |
|  | 45 | ʟ-Sorbose | ᴅ-Sorbitol |  | One of the top sustainable platform chemicals | (Adachi et al., 1999a) |
|  | 46 | ʟ-Sorbose | 2-Keto-ʟ-gulonic acid |  | The precursor of vitamin C | (Sugisawa et al., 1991) |
|  | 47 | Galactose | Galactonic acid |  | -- | (Švitel and Šturdik, 1994) |
|  | 48 | 2-Deoxy-ᴅ-ribose | 2-Deoxy-4-keto-ᴅ-ribose |  | -- | (Adachi et al., 2013) |
|  | 49 | 2-Deoxy-ᴅ-ribose | 2-Deoxy-4-Keto-ᴅ-ribonate |  | -- | (Adachi et al., 2013) |
|  | 50 | Lactose | Lactobionic acid |  | Used in food processing, detergents, medicine and fine chemical industries | (Kiryu et al., 2014) |
|  | 51 | Isomaltose | Isomaltobionic acid |  | Potential health applications in food | (Kiryu et al., 2020) |
|  | 52 | Melibiose | Melibionic acid |  | Potential health applications in food | (Kiryu et al., 2020) |
|  | 53 | Gentiobiose | Gentiobionic acid |  | Potential health applications in food | (Kiryu et al., 2020) |
| AOF products from sugar alcohols | 54 | ᴅ-Sorbitol | ʟ-Sorbose |  | Intermediate of vitamin C production | (Sato et al., 1967 ; Cummins et al., 1957) |
|  | 55 | ᴅ-Sorbitol  ᴅ-Mannitol | ᴅ-Fructose |  | Biochemical and microbiological research, a medicinal and sweetener | (Cummins et al., 1957; Adachi et al., 1999b) |
|  | 56 | ᴅ-Sorbitol | 5-Keto-ᴅ-fructose |  | -- | (Sato et al., 1967) |
|  | 57 | ᴅ-Sorbitol | Kojic acid |  | Inhibit the synthesis of tyrosinase, as food additives | (Sato et al., 1967) |
|  | 58 | ᴅ-Sorbitol | 3-Oxykojic acid |  | -- | (Sato et al., 1967) |
|  | 59 | ᴅ-Sorbitol | 5-Oxymaltol |  | -- | (Sato et al., 1967) |
|  | 60 | Glycerol | Glyceraldehyde |  | Intermediate products of carbohydrate metabolism | (Habe et al., 2009) |
|  | 61 | Glycerol | Glyceric acid |  | Food additives, textile softener, drug transports carrier, etc. | (Habe et al., 2009) |
|  | 62 | Glycerol | Dihydroxyacetone |  | The active ingredient in sunscreen products | (Habe et al., 2009 ; Hu et al., 2010) |
|  | 63 | Racemic glycidol | Glycidic acid |  | Photorespiratory inhibitor | (Švitel and Kutnik, 1995) |
|  | 64 | ʟ-Fucitol | ʟ-Duco-4-ketose |  | -- | (Richtmyer et al., 1950) |
|  | 65 | Meso-erythritol | ʟ-Erythrulose |  | Important intermediate and multi-performance additive in chemical synthesis | (Richtmyer et al., 1950) |
|  | 66 | ᴅ-Arabitol | ᴅ-Xylulose |  | For xylitol production | (Suzuki et al., 2002) |
|  | 67 | ᴅ-Arabitol | Xylitol |  | Food sweetener | (Suzuki et al., 2002) |
|  | 68 | Allitol | ʟ-Psicose |  | Only used for experimental studies such as content determination | (Takeshita et al., 1996 ; Carr et al., 1968) |
|  | 69 | Ribitol | ʟ-Ribulose |  | Standard for chemical analysis of sugars | (Adachi et al., 2001) |
|  | 70 | Galactitol | ᴅ-Tagatose |  | A low-calorie sweetener | (Xu et al., 2021) |
|  | 71 | Galactitol | ʟ-Xylo-3-hexulose |  | A healthy alternative to traditional sugar and sweeteners | (Xu et al., 2021) |
| AOF products from acids | 72 | Dihydroshikimic acid | 3,4-Dihydroxy-5-oxoxylohexane-1-carboxylic acid |  | -- | (Whiting and Coggins, 1967) |
|  | 73 | Quinate | 3-Dehydroquinate |  | Intermediate of shikimic acid pathway | (Adachi et al., 2006) |
|  | 74 | Quinate  shikimate | 3-Dehydroshikimate |  | Intermediate of shikimic acid pathway | (Adachi et al., 2006) |
|  | 75 | Quinate | Protocatechuate |  | Antibacterial, anti-oxidation, anti-cancer, etc. | (Adachi et al., 2006) |
|  | 76 | Malate | Oxaloacetate |  | An intermediate in the citric acid cycle | (Benziman and Perez, 1965) |
|  | 77 | Ca-Mg lactate | Ca-Mg acetate |  | A potentially non-corrosive and biodegradable deicing chemical | (Dosoretz et al., 1992) |
|  | 78 | ʟ/ᴅ-Lactate | Pyruvate |  | An edible spice and used in the synthesis of medicine, amino acids, etc. | (Sato et al., 2015) |
| AOF products from others | 79 | ᴅ-Glucosamine | ᴅ-Glucosaminic acid |  | An important precursor in glycosylation reaction of proteins or lipids | (Takahashi and Kayamori, 1960) |
|  | 80 | ᴅ-Mannosamine | ᴅ-Mannosaminate | -- | -- | (Moonmangmee et al., 2004) |
|  | 81 | ᴅ-Galactosamine | ᴅ-Galactosaminate | -- | -- | (Moonmangmee et al., 2004) |
|  | 82 | Furfural alcohol | 2-Fruoic acid |  | Used as plasticizer, thermosetting resin, preservative and coating additive, etc. | (Zhou et al., 2017) |
|  | 83 | 5-Hydroxymethyl furfural | 5-Hydroxymethyl-2-furan-carboxylic acid |  | As a therapeutic based on its antimicrobial and antitumor activity | (Sayed et al., 2019) |
|  | 84 | ʟ-Gulono-γ-lactone | ʟ-Ascorbic acid |  | Water-soluble vitamin, used in cosmetics, medicine | (Sugisawa et al., 2005) |
|  | 85 | N-Butylglucamine | 6-Deoxy-6-butylaminosorbose |  | Preparation of N-Butyldeoxynojirimycin | (Landis et al., 2002) |
|  | 86 | N-2-Hydroxyethyl glucamine | 6-(2-Hydroxy-ethyl) amino-6-deoxy-α-ʟ-sorbofuranose |  | Precursor, key intermediate of miglitol | (Keliang and Dongzhi, 2006) |

*: The researchers speculated the results.

--: No relevant structures or functions have been collected.
